# Circulating and tissue biomarkers in early-stage non-small cell lung cancer

**DOI:** 10.3332/ecancer.2017.717

**Published:** 2017-01-31

**Authors:** Caterina Fumagalli, Fabrizio Bianchi, Paola Rafaniello Raviele, Davide Vacirca, Giovanni Bertalot, Cristiano Rampinelli, Matteo Lazzeroni, Bernardo Bonanni, Giulia Veronesi, Nicola Fusco, Massimo Barberis, Elena Guerini-Rocco

**Affiliations:** 1 Division of Pathology, European Institute of Oncology, Via Giuseppe Ripamonti 435, 20141, Milan, Italy; 2 Institute for Stem-cell Biology, Regenerative Medicine and Innovative Therapies (ISBReMIT), IRCCS Casa Sollievo della Sofferenza, Viale Cappuccini 1, 71013, San Giovanni Rotondo, Foggia, Italy.; 3 Molecular Medicine Programme IEO, European Institute of Oncology, Via Giuseppe Ripamonti 435, 20141, Milan, Italy; 4 Department of Radiology, European Institute of Oncology, Via Giuseppe Ripamonti 435, 20141, Milan, Italy; 5 Division of Cancer Prevention and Genetics, European Institute of Oncology, Via Giuseppe Ripamonti 435, 20141, Milan, Italy; 6 Division of Thoracic Surgery, Humanitas Research Hospital, Via Manzoni 56, 20089, Rozzano Milan, Italy; 7 Division of Pathology, Fondazione IRCCS Ca’ Granda – Ospedale Maggiore Policlinico, University of Milan, Via Francesco Sforza 35, 20122, Milan, Italy

**Keywords:** non-small cell lung cancer, early detection, circulating biomarker, somatic mutation

## Abstract

**Objective:**

We sought to characterise circulating and tissue tumour biomarkers of patients who developed early-stage non-small cell lung cancer (NSCLC) during long-term follow-up of a chemoprevention trial (NCT00321893).

**Materials and Methods:**

Blood and sputum samples were collected from 202 high-risk asymptomatic individuals with CT-detected stable lung nodules. Real-time PCR was performed on plasma to quantify free circulating DNA. Baseline serum was investigated with a previously validated test based on 13 circulating miRNAs (miR-Test). Promoter methylation status of p16, RASSF1a and RARβ2 and telomerase activity were assessed in sputum samples. DNA was extracted from each tumour developed during follow-up and subjected to a mutation survey using the LungCarta panel on the Sequenom MassARRAY platform.

**Results:**

During follow-up (9 years) six individuals underwent surgery for stage I NSCLC with a median time of disease onset of 20.5 months. MiR-Test scores were positive (range: 0.14–7.24) in four out of six baseline pre-disease onset sera. No association was identified between free circulating DNA or sputum biomarkers and disease onset. All tumours harboured at least one somatic mutation in well-known cancer genes, including *KRAS* (n = 4), *BRAF* (n = 1), and *TP53* (n = 3).

**Conclusion:**

Circulating miRNA tests may represent valuable tools to detect clinically-silent tumours. Early-stage lung adenocarcinomas harbour recurrent genetic events similar to those described in advanced-stage NSCLCs.

## Introduction

Non-small cell lung cancer (NSCLC) is the leading cause of cancer-related deaths worldwide [1]. Primary prevention based on tobacco control programmes still remains the most effective approach to tackle this highly lethal disease. However, early detection of lung cancer represents a fundamental strategy to reduce disease-associated mortality and allowing for the application of potentially curative treatments (*i.e.* surgical resection) [2]. Unfortunately, only less than 20% of patients are currently diagnosed with a locally confined disease [3].

Over the past decade, low-dose computed tomography (LDCT) has been shown to be an effective screening tool for early detection of tumours in high-risk populations, reducing lung cancer mortality [4, 5]. However, the majority of the LDCT-detected lung nodules are benign lesions [5, 6], entailing further expensive and invasive procedures. In this scenario, circulating biomarkers might represent the ultimate complementary tools to improve the cost-effectiveness of screening protocols in high-risk populations, decreasing the number of false positive cases, and allowing for non-invasive early diagnosis [7]. Circulating-free DNA (cfDNA) has been extensively investigated as a potential diagnostic, predictive, and prognostic non-invasive biomarker in different tumour types [8–10]. Quantitative and qualitative alterations of cfDNA, including both genetic and epigenetic aberrations, have been described in blood and sputum samples from patients with NSCLCs even at early stages of tumour onset [11–16]. Moreover, the expression profiles of circulating short non-coding microRNA molecules (miRNAs) have been shown to represent a compelling non-invasive biomarker for the diagnosis of cancer [17, 18]. Recently, different studies have reported on the accuracy of different serum and plasma circulating miRNA signatures for early detection of lung tumour [19–23]. In particular, Montani *et al* identified a serum miRNA signature (miR-Test) based on 13 circulating miRNAs which have been validated in previous lung cancer screening studies demonstrating a high sensitivity and specificity (77.8% and 74.8%, respectively) for early diagnosis of lung cancer [22]. In addition, chemoprevention strategies have been combined with smoking cessation and screening programmes using agents, such as corticosteroids or aspirins, with heterogeneous and still non-conclusive results about their effect of lung cancer risk-reduction [24–26].

In this study, we sought to explore the landscape of circulating and tumour tissue molecular biomarkers of patients who developed early-stage NSCLC during long-term follow-up of randomised, double-blind, phase IIb chemoprevention trial nested in a computed tomography (CT)-scan lung cancer screening programme (NCT00321893) [24].

## Materials and methods

### Population and samples

Study design and eligibility criteria of the randomised, double-blind, phase IIb chemoprevention trial (NCT00321893) have been extensively described before [27]. Briefly, 202 high-risk asymptomatic subjects, current or former smokers, with CT-detected lung nodules that were persistent for at least one year were randomised to receive budesonide (800 µg) or placebo twice daily for 12 months. Nodule types were classified as non-solid (n = 23), partially solid (n = 41), solid (n = 184), and sub-solid (n = 64). Sputum and blood samples were collected from each individual at baseline and after 6 and 12 months of treatment. Both plasma and serum were obtained from blood samples. Informed consent was obtained as specified in the trial protocols and previously reported [24, 27]. Dropout rate was 2%. Clinical short-term and long-term effects of one year of inhaled budesonide on screening-detected lung nodules have already been reported at a follow-up of one and five years respectively [24, 28]. During follow-up (nine years in total), six patients developed NSCLC.

### Circulating biomarker analyses

Promoter methylation status of *p16^INK4A^*, *RASSF1a*, and *RARβ2* genes was analysed in sputum samples with quantitative methylation specific PCR (QMSP) as previously described [16, 29, 30]. Telomerase activity assay was performed on sputum using commercial available kit (TeloTAGGG Telomerase PCR ELISA^PLUS^, Roche) following manufacturer’s protocol. Sputum analyses were performed at baseline and after 12 months from enrolment. Using a real-time quantitative PCR, cfDNA was quantified on plasma samples collected at baseline after 6 and later 12 months from enrolment according to Sozzi *et al* [31]. The previously validated miR-Test was retrospectively assessed on baseline serum of the six patients who developed NSCLC and fifty cancer-free individuals enrolled in the same trial. Serum miRNA purification and expression profiling were performed according to Montani *et al* [22]. Briefly, total RNA purification, including miRNAs, was based on lysis with guanidinium thiocyanate-phenol-chloroform extraction (TriZol-LS, Applied Biosystem) and Spin Column-based total RNA purification (MiRneasy Mini Kit, Qiagen). MiRNA qRT-PCR was carried out on a ViiA™ 7 instruments (ThermoFisher) using the manufacturer’s recommended cycling conditions. MiRNA qRT-PCR data were automatically analysed using a custom R script that provides miR-Test risk scores automatically. Patients were classified as ‘positive’ or ‘negative’ for the miR-Test based on a risk score ≥ 0 or < 0 respectively [22].

### Tumours pathologic assessment and mutation analysis

Haematoxylin and eosin stained sections of each case were reviewed by two pathologists; tumours were staged and subtyped according to WHO Classification of Tumours of the Lung [32]. DNA was extracted from representative 5-*µ*m-thick sections cut from formalin-fixed and paraffin-embedded blocks of each tumour sample to ensure tumour cell content is above 20% as previously described [33]. Genomic DNAs were subjected to a mutation survey using the LungCarta panel (Sequenom), including evaluation of 214 somatic mutations in 26 oncogenes and tumour suppressor genes, and analysed on a matrix-assisted laser desorption/ionisation time-of-flight (MALDI-TOF) mass spectrometer (Sequenom) following manufacturer’s protocol. Data were evaluated using MassARRAY TYPER ANALYSER software 4.0, with a limit of detection of 5%.

## Results

**Figure 1.**
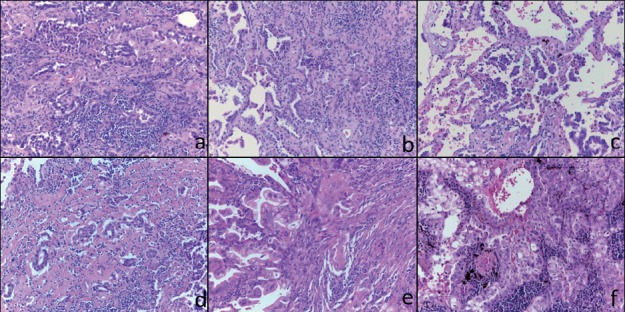
Histological features of the six tumours included in this study. Representative micrographs of the six tumours showing adenocarcinoma with a prevalent lepidic and/or acinar growth pattern (a-f) with focal formation of micropapillae in case 3 (c) and solid nests in case 6 (f). Haematoxylin and eosin-stained slides; original magnification 200x.

No tumour occurred during the 12 months of trial treatment. Only six patients, three in the budesonide arms and three in the placebo arms, were diagnosed with NSCLC during the following 9 years of follow-up with a median time from enrolment to disease onset of 663.5 days. One of the six patients underwent a local pulmonary recurrence 3 years after the first diagnosis. After a median follow-up of 2104 days from surgery, each patient was alive and disease-free. Patient’s characteristics are reported in Table 1. All tumours were classified as stage I lung adenocarcinomas (ADC) including one case with multifocal minimally-invasive adenocarcinoma (MIA). All lesions displayed a prevalent lepidic and/or acinar pattern of growth (Figure 1). The clinic-pathologic characteristics of tumours and the radiologic features of nodules at baseline and at the time of tumour onset are summarised in Table 1.

No significant differences were observed in plasma cfDNA or sputum biomarkers between patients who developed NSCLC and the whole trial cohort (Table 2). The epigenetic analysis revealed *p16^INK4A^* or *RASSF1a* methylation in 16 samples and none from patients who developed lung cancer.

Interestingly, telomerase activity was negative in all but two sputum samples collected at 12 months after the beginning of the treatment. Both samples were obtained from two of the patients who subsequently developed lung cancer (patients #1 and #3) (Table 2), and the presence of telomerase activity was also confirmed in their corresponding frozen tumour tissues.

The miR-Test revealed a positive score in four out of six baseline pre-disease onset sera (sensitivity 67%) of patients that subsequently were diagnosed with NSCLC (median: range: +0.14–+4.81). Remarkably, the two miR-Test negative patients showed a positive telomerase activity in their sputum samples. Among the 50 cancer-free individuals, 36 had a negative miR Test score (specificity 72%), with a median risk score of -6.2 (range: -21.5 – +28.9) (Table 2).

All tumour samples harboured at least one driver somatic mutation in well-known cancer-related genes, including *KRAS* (n = 4), *BRAF* (n = 1), and *TP53* (n = 3) (Table 3). Interestingly, non-synonymous somatic missense mutations of *KRAS* were detected in four out of the six (66.67%) early-stage adenocarcinoma analysed with a frequency even higher than that described in early and advanced-stage adenocarcinoma of high-risk individuals (32% and 35% respectively) [34–36].

## Discussion

The continuous monitoring of high-risk individuals enrolled in lung cancer-screening programmes represents a great opportunity not only in detecting early-stage, potentially curable disease but also in validating and/or identifying new diagnostic molecular biomarkers of lung cancer. Here, we performed a multi-level characterisation of circulating and tumour tissue biomarkers of a unique group of patients who developed early-stage NSCLCs during long-term follow-up of a chemoprevention trial (NCT00321893) [24] nested in CT-scan screening programme [37].

Primary and secondary prevention strategies still remain the pivotal steps to reduce the high-rate of NSCLC-associated mortality. Over the past decade, many efforts have been made to identify effective programmes of prevention for high-risk individuals, including chemoprevention and imaging screening studies. In this study, among 202 high-risk asymptomatic subjects with CT-detected lung nodules enrolled in one-year budesonide-based chemoprevention trial, only six patients developed NSCLC during long-term follow-up with a rate slightly lower than that described in the entire nested screening programme population (3% versus 5.7%) [38]. It should be noted that these rates refer specifically to the follow-up timeframe of this study. As previously reported [24, 28], no differences were identified in the distribution of lung cancer between treatment- and placebo-arm. All patients were diagnosed with early-stage NSCLCs and were eligible for surgical resection confirming the efficacy of such screening programmes in the early detection of potentially curable diseases.

The median concentration of plasma cfDNA and sputum *RARβ2* gene methylation did not show any statistically significant difference in NSCLC patients compared to the cancer-free group. Moreover, no gene promoter methylation of *p16^INK4A^* and *RASSF1a* was detected in any sputum sample from lung-cancer patients. Furthermore, four tumours harboured *KRAS* somatic mutations that have been previously shown to have a tendency toward mutual exclusivity with *RASSF1a* promoter methylation in colorectal and non-small cell lung cancers [39, 40]. These results suggest that plasma cfDNA and sputum gene methylation quantifications, albeit specific [16], are likely to represent low-sensitive diagnostic biomarkers, especially in NSCLC-screening settings.

The sputum telomerase activity assay was able to capture two out six clinically-silent lung tumours. In previous studies, tissue telomerase activity has been correlated with poor-prognostic early and advanced-stage NSCLCs [15, 41]. The identification of telomerase activity in pre-disease onset sputum indicates that it might represent also a potentially useful diagnostic biomarker in early-stage lung cancers. Interestingly, Ilie *et al* described a sensitive circulating tumour cell (CTC) detection approach to identify patients 'at risk' of developing lung cancer before any clinically detectable CT scan nodules [42]. Additional multicentric studies are warranted to define the specificity and sensitivity of this approach in high-risk asymptomatic subjects with CT-detected lung nodules and to characterise the potential role of these circulating biomarkers as reliable screening tools for early detection of lung cancer in clinical practice.

Since the design of this trial protocol [27] new circulating biomarkers for early-detection of NSCLC have been investigated. In particular, the miR-Test has been recently described as a promising tool to detect clinically-silent tumours [21]. Moreover, this test has been already validated in different sets of high-risk asymptomatic individuals enrolled in CT-scan screening programmes [22]. In our study, the miR-Test showed a positive score in the majority of patients who subsequently were diagnosed with NSCLCs, with an accuracy of 71% (sensitivity 67%; specificity 72%). These values were slightly lower than those reported in the study by Montani *et al* (sensitivity and specificity of 77.8% and 74.8% respectively) [22]. These differences may be related to the small number of tumours observed in our study group that prevented any predictive value analysis. On the other hand, the routinely assessment of the miR-Test in different populations may strengthen the previous validated results. Indeed, a new chemoprevention trial has been already designed including the miR-Test as a predictive diagnostic biomarker [43]. Interestingly, the two patients with negative miR-Test scores showed positive telomerase activity in their sputum samples. This observation seems to suggest that the combined use of these two molecular biomarkers might be able to improve the risk assessment of NSCLC development in high-risk populations.

The usefulness of molecular genotyping of early-stage NSCLC remains controversial. Conflicting results have been reported on the predictive and prognostic role of the mutations identified in early-stage disease including *EGFR* and *KRAS* mutations [44–46]. However, molecular analysis of early-stage NSCLC may constitute the substrate not only for the implementation of personalised therapies but also for the identification of new diagnostic molecular biomarkers. In this study, all cases harboured somatic mutations in at least one cancer-related gene that likely represents the driver of these tumours. Notably, the patients were all current or former smokers and in four out of six tumours we identified somatic mutations of *KRAS* that were frequently associated with the carcinogenic effect of tobacco smoke [44]. Moreover, the incidence of *KRAS* aberrations was even higher than that described in advanced-stage NSCLC (67% versus 35%) [34–36].

Interestingly, no mutations were identified in *EGFR* gene that together with *KRAS* is the most commonly mutated gene in lung adenocarcinoma. Furthermore, we detected one rare somatic mutation of *BRAF* and three *TP53* somatic mutations that have been previously described in nearly 45% of early-stage adenocarcinoma of high-risk patients [34–36]. Although a sample-size bias cannot be excluded, these significant inter-patient differences in gene mutation frequencies may be related to the peculiar clinical characteristics of the study group (*i.e.* high-risk individuals with current or former smoking history and lung nodules).

The small size of the invasive or minimally invasive adenocarcinomas of this series shows that genetic aberrations driving tumour progression develop very early in the tumourigenesis. This finding has been already observed in atypical adenomatous hyperplasia that represents the morphologic continuum ending in the full-blown adenocarcinoma. Previous studies have demonstrated that atypical adenomatous hyperplasia can harbour some of the genetic alterations found in adenocarcinomas, including mutations of *KRAS, EGFR, and TP53* [47–49]. The presence of driver genetic events even at early stage of tumour development offers the opportunity to test these mutations in cfDNA as potential biomarkers for early detection of lung cancer. Recently, the study of Izumchenko *et al* provided the proof-of-concept that genetic alterations associated with very small early glandular neoplasms can be detected in paired circulating DNA even before they invade and acquire malignant potential [50]. Molecular profiling of cfDNA is emerging as key non-invasive tool for monitoring tumour progression and therapy response/resistance. However, concerns about the specificity and sensitivity of this assays in the screening setting have so far limited its clinical application [8, 51]. Indeed, the relatively high sensitivity of cfDNA hotspot mutation analysis tests (e.g. digital PCR) requires a prior knowledge of the tumour mutational profile which pose a major limitation of its use for screening purpose [52]. It should be noted, however, that next generation sequencing (NGS) technologies allow for simultaneous assessment of multiple genetic aberrations in ‘unsupervised’ manner [52, 53]. These high-throughput assays have recently shown the ability to detect cfDNA mutations even in early-stage lung tumours [52, 54, 55]. These pioneer results may pave the way for future application of NGS-based cfDNA analysis as potential biomarker for lung cancer screening.

Our study has several limitations, including the small sample size, which precluded statistical subgroup analyses. Indeed, given the specific primary endpoint of the NCT00321893 trial, the sample size was not powered to define the statistically significance of circulating biomarkers and early detection of lung cancer. In addition, this is a retrospective study performed on samples prospectively collected before the trial completion date. For this reason, sputum and blood samples at the time of cancer diagnosis were not available. However, this is an exploratory analysis of potential circulating biomarkers of lung cancer development in a unique prospective cohort of high-risk individuals enrolled in a lung cancer chemoprevention trial. The new era of screening studies will be grounded in multidisciplinary strategies including clinical information, imaging approaches, and circulating biomarkers analyses that might allow for non-invasive highly sensitive and specific early detection of lung cancer.

## Conclusion

In conclusion, our exploratory study confirms the pivotal role of screening programmes and highlights the clinical value of circulating biomarkers, including miRNA and sputum telomerase activity tests, in detecting clinically-silent early-stage NSCLC. Moreover, we corroborate the notion that even clinically and histologically early lung adenocarcinoma may be underpinned by somatic genetic events similar to those described in advanced-stage NSCLCs.

## Funding

The NCT00321893 trial was supported by the National Cancer Institute Division of Cancer Prevention, contract N01-CN-035159 to the UT MD Anderson Early Phase Chemoprevention Consortium.

## Financial disclosures

FB is an inventor on a patent application regarding a diagnostic serum miRNA signature cited herein.

## Figures and Tables

**Table 1.** Clinico-pathologic features of baseline nodules and tumors of the six patients who developed lung adenocarcinoma.

Patients ID	Gender	Smoking History	Treatment	Baseline nodules			Time from Randomization to Surgery	Tumors							
				Site	Type	CT-dimension (mm)		Site	CT-dimension (mm)	Histotype		Grading	Stage		
													T	N	M
1	Male	current smoker	placebo	RSL	partially solid	6.4	567	RSL	8	ADC	lepidic/acinar	2	1a	0	0
2	Male	former smoker	placebo	RLL	na	na	2811	RLL	12	MIA	lepidic	1	1a(m)	0	0
3	Male	current smoker	budesonide	RLL	partially solid	5.2	468	RLL	9	ADC	lepidic/micropapillary	2	1a	0	0
4	Male	current smoker	budesonide	RSL	solid	2.7	425	RSL	6	ADC	acinar/solid	3	2a	0	0
5	Male	former smoker	placebo	RLL	partially solid	5.1	760	RLL	7	ADC	mucinous	1	1a	0	0
6	Female	former smoker	budesonide	RSL	partially solid	5	1649	RSL	8	ADC	solid/lepidic	2	1a	0	0
RSL, right superior lobe; RLL, right lower lobe; CT, computer tomography; na, not available; ADC, adenocarcinoma; MIA, minimally invasive adenocarcinoma.															

**Table 2.** Circulating biomarkers of the six patients who developed lung adenocarcinoma and median values of the lung cancer-free group.

Patients ID	RARB2 methylation (%)		Telomerase activity		cfDNA (ng/ml)			miR Test	
	Sputum		Sputum		Plasma			Baseline Serum	
	Baseline	After 12 months	Baseline	After 12 months	Baseline	After 6 months	After 12 months	Score	Neg/Pos
1	0.814	0.52	Negative	Positive	0.193	0.194	1.034	-2.81	Negative
2	2.169	7.595	Negative	Negative	11.952	3.054	19.936	0.34	Positive
3	0.553	0	Negative	Positive	0.309	1.577	4.15	-6.59	Negative
4	1.63	2.613	Negative	Negative	7.453	1.917	2.694	0.14	Positive
5	0.083	3.812	Negative	Negative	4.975	9.79	16.138	1.34	Positive
6	0.075	0.534	Negative	Negative	5.08	0.412	2.302	4.81	Positive
**patients group (n = 6) median values**	0.6835	1.5735	Negative	na	5.0275	1.747	3.422	0.24	na
**control group (n = 96) median values**	0.439	0.853	Negative	Negative	4.271	3.994	3.328	-6.2#	na

na, not available; # control group of miR-Test n = 50.

Promoter methylation status of RARβ2 and telomerase activity assays were performed on sputum samples collected at baseline and after 12 months from enrollment. cfDNA was quantified on plasma samples collected at baseline and after 6 and 12 months from enrollment. MiR-Test was performed on baseline serum.

**Table 3.** Non-synonymous somatic mutations identified in the six lung adenocarcinoma.

Patients ID	Non-synounymous somatic mutation
1	*TP53* p.R248L c. 743G>T
2	*BRAF* p.G466V c.1397G>T; *TP53* p.D281E c.843C>G
3	*KRAS* p.G13E c.38_39GC>AA
4	*KRAS* p.G12V c.35G>T; *TP53* p.Y234C c.701A>G
5	*KRAS* p.G12V c.35G>T
6	*KRAS* p.G12D c.35G>A
